# Management of Traumatic Arterial Injuries of the Hand and Digits in the Emergency Department: A Systematic Review

**DOI:** 10.7759/cureus.117758

**Published:** 2026-10-11

**Authors:** Mazin Osman, Syeda Maida Mujahid, Osama Ali, Shenouda R Shehata Abdelmesih, Saad Ali, Shashwat Shetty, Hira Khan

**Affiliations:** 1 General Surgery, British United Provident Association, Jeddah, SAU; 2 Orthopaedics and Traumatology, Luton and Dunstable Hospital, Luton, GBR; 3 Emergency Medicine, University Hospital Limerick, Limerick, IRL; 4 Orthopaedics and Trauma, Royal Gwent Hospital, Newport, GBR; 5 Emergency Medicine, Nishtar Hospital, Multan, PAK; 6 Orthopaedics, Hillingdon Hospital, Uxbridge, GBR; 7 Medicine, Jinnah Sindh Medical University, Karachi, PAK

**Keywords:** arterial injury, digital artery, hand trauma, revascularisation, traumatic hand injury

## Abstract

Traumatic arterial injuries of the hand are uncommon but may threaten digit viability and function, while the optimal indications for vascular reconstruction remain uncertain. A systematic literature search of PubMed or MEDLINE, Scopus, Embase and Cochrane Library was performed between 2 April and 30 July 2026 in accordance with Preferred Reporting Items for Systematic Reviews and Meta-Analyses (PRISMA) 2020. Studies evaluating traumatic arterial injuries confined to the hand and digits were eligible. Of 98 records identified, six studies met the inclusion criteria, comprising approximately 546 patients across the included cohorts. Evidence supported revascularisation of threatened or ischaemic digits, with primary repair preferred when a tension-free anastomosis was achievable and interposition vein grafting used for segmental defects. In adequately perfused digits, isolated digital arterial injury did not consistently require reconstruction. Dual arterial reconstruction improved objective perfusion but did not demonstrate a consistent functional advantage, while artery-only replantation showed acceptable survival in selected distal amputations. Management should be individualised according to perfusion, mechanism and extent of arterial injury. Current evidence is predominantly observational and heterogeneous, supporting the need for prospective multicentre comparative studies.

## Introduction and background

Traumatic injuries of the hand are among the commonest upper extremity injuries presenting to emergency departments. In a US population-based analysis of emergency department presentations, approximately 3.47 million upper extremity injuries were estimated to occur in 2009, corresponding to an annual injury rate of 1,130 cases per 100,000 people; the fingers were the most frequently injured anatomical region, accounting for 38.4% of upper extremity injuries. Finger lacerations alone occurred at an estimated rate of 221 per 100,000 persons per year [1]. However, the epidemiology of isolated traumatic arterial injury of the hand and digits is considerably less well characterized, reflecting its relative rarity and the tendency of trauma registries to group vascular injuries across the entire upper extremity. Upper extremity vascular injuries constitute a small proportion of overall trauma but can result in substantial morbidity when arterial perfusion is compromised [2]. The clinical importance of hand arterial trauma arises from the complex and variable collateral circulation of the hand. The superficial and deep palmar arches and their digital branches provide extensive arterial anastomoses, allowing some patients to maintain adequate tissue perfusion despite disruption of an individual arterial vessel. Consequently, the identification of an arterial injury does not necessarily indicate critical ischaemia or mandate immediate arterial reconstruction. Conversely, disruption of multiple arterial channels, traumatic amputation, crush or avulsion injury, or extensive arterial wall damage may result in threatened digital viability. This distinction makes early physiological assessment particularly important in the emergency department, where clinical examination, capillary refill, skin temperature and colour, bleeding, palpable pulses and Doppler signals are used to establish the adequacy of distal perfusion [3]. 

The management of traumatic arterial injury therefore requires a balance between rapid recognition of threatened tissue and avoidance of unnecessary vascular intervention in a well-perfused hand or digit. Non-invasive vascular assessment, including Doppler ultrasonography, may provide additional anatomical and haemodynamic information when the clinical examination is equivocal, while angiographic imaging can be considered when the extent or location of vascular injury remains uncertain. The broader vascular trauma literature supports prompt identification and treatment of clinically significant arterial injury, while recognising that management should be individualised according to mechanism, anatomical level, collateral circulation and associated soft tissue, tendon, nerve, and skeletal injury [4]. The available literature also demonstrates that upper extremity vascular injury encompasses a heterogeneous group of lesions, meaning that evidence derived from brachial, forearm and proximal arterial trauma cannot necessarily be extrapolated directly to the unique vascular anatomy and functional requirements of the hand.

Despite the clinical importance of these injuries, there is still no clearly proven, hand-specific evidence-based pathway integrating emergency assessment, vascular imaging, and definitive arterial treatment. Furthermore, the evidence concerning when an injured digital artery requires reconstruction, when primary repair should be replaced by an interposition graft, and whether restoration of one or multiple digital arterial channels improves clinical outcomes remains heterogeneous. The primary aim of this systematic review was to evaluate the surgical management of traumatic arterial injuries of the hand and digits, including arterial repair, interposition vein grafting, revascularisation and replantation. Secondary objectives were to examine factors associated with treatment success, arterial patency and digit survival, as well as sensory and functional outcomes and complications following different reconstructive strategies.

## Review

Materials and methods

Search Strategy

A systematic search of the literature was performed across PubMed, MEDLINE, Embase, Scopus, and the Cochrane Library, covering all records available from inception, with the initial search conducted on 2 April 2026 and the final search conducted on 30 July 2026. The search strategy was structured around three core concepts: anatomical site, traumatic arterial injury, and assessment and treatment, using controlled vocabulary (e.g., MeSH and database-specific subject headings) combined with relevant free text terms such as hand, finger, digit, palmar artery, digital artery, arterial injury, vascular injury, hand ischaemia, trauma, laceration, amputation, revascularisation and replantation. Boolean operators (AND and OR), truncation techniques, and database-specific search syntax were employed to enhance the sensitivity and reproducibility of the search strategy. In addition, the reference lists and citation networks of the included studies and relevant systematic reviews were examined to identify further eligible publications. The review was reported in accordance with the Preferred Reporting Items for Systematic Reviews and Meta-Analyses (PRISMA) 2020 guidelines. A PRISMA 2020 flow diagram was used to provide a transparent account of the search process, including records identified, duplicates removed, records screened, full-text articles assessed for eligibility, reasons for exclusion, and studies ultimately included in the review [5]. The complete database-specific search strategies are provided in Table 1.

**Table 1:** Complete Database-Specific Search Strategies

Database	Date searched	Search strategy
PubMed/MEDLINE	2 April 2026; final search 30 July 2026	(“Hand” OR “Finger” OR hand* OR finger* OR digit*) AND (“Arteries” OR arter* OR vascular*) AND (“Wounds and Injuries” OR trauma* OR injur* OR lacerat* OR amputat*) AND (revasculari* OR replant* OR repair* OR reconstruction* OR “vein graft” OR graft*)
Embase	2 April 2026; final search 30 July 2026	(“hand” OR “finger” OR hand* OR finger* OR digit*) AND (“artery” OR arter* OR vascular*) AND (“trauma” OR “wound” OR trauma* OR injur* OR lacerat* OR amputat*) AND (revasculari* OR replant* OR repair* OR reconstruction* OR “vein graft” OR graft*)
Scopus	2 April 2026; final search 30 July 2026	(hand* OR finger* OR digit*) AND (arter* OR vascular*) AND (trauma* OR injur* OR lacerat* OR amputat*) AND (revasculari* OR replant* OR repair* OR reconstruction* OR “vein graft” OR graft*)
Cochrane Library	2 April 2026; final search 30 July 2026	(“Hand” OR “Finger” OR hand* OR finger* OR digit*) AND (“Arteries” OR arter* OR vascular*) AND (“Wounds and Injuries” OR trauma* OR injur* OR lacerat* OR amputat*) AND (revasculari* OR replant* OR repair* OR reconstruction* OR “vein graft” OR graft*)

Eligibility Criteria

Studies were considered eligible according to a predefined PICO (Population, Intervention, Comparator and Outcomes) framework addressing the assessment and treatment of traumatic arterial injuries of the hand [6]. The population (P) comprised adult or paediatric patients with traumatic arterial injuries involving the hand or digits, including injuries associated with amputation, revascularisation or replantation. Interventions (I) included primary arterial repair, interposition vein grafting, arterial reconstruction, revascularisation, and replantation techniques. Comparators (C) included alternative treatment strategies, such as primary repair versus grafting, reconstruction versus nonreconstruction, single versus dual arterial reconstruction, and conventional versus artery-only replantation. Studies without formal comparators were also eligible if clinically relevant outcomes were reported. Outcomes (O) included arterial patency, digit survival, treatment success, complications, reoperation, amputation, sensory and functional outcomes, pain and cold intolerance. Studies focusing exclusively on arm or forearm injuries were excluded, as were animal and cadaveric studies, review articles, editorials, letters, conference abstracts, and case reports. Duplicate publications were identified and managed to avoid double counting. Also, Studies published in languages other than English were excluded.

Study Selection

All retrieved records were transferred to a reference management software, where duplicate entries were identified and removed prior to the screening process. Two reviewers independently assessed the titles and abstracts according to the predefined eligibility criteria. Articles deemed potentially eligible were subsequently evaluated through full-text review, with reasons for exclusion recorded systematically. Discrepancies between the reviewers were first resolved through discussion and, when agreement could not be achieved, were adjudicated by a third reviewer.

Data Extraction

Data were extracted independently by two reviewers using a predefined and piloted data extraction form. Extracted variables included author and year of publication, country, study design, sample size, patient demographics, mechanism of injury, anatomical level and type of arterial injury, presence and severity of hand or digital ischaemia, associated nerve, tendon or bone injuries, vascular assessment methods, imaging techniques, operative treatment, primary arterial repair, interposition vein grafting, single versus dual arterial reconstruction, revascularisation or replantation techniques, and comparator interventions. Outcome data included arterial patency, digit or hand survival, revascularisation success, complications, reoperation, amputation, sensory recovery, cold intolerance, pain, range of motion, grip strength, functional scores, return to work and quality of life outcomes. Follow-up duration and loss to follow-up were also recorded. Disagreements in extracted data were resolved by consensus or third reviewer adjudication.

Risk of Bias Assessment

Risk of bias was assessed using the Joanna Briggs Institute (JBI) critical appraisal tools appropriate to the design of each included study [7]. The appraisal findings were considered during interpretation of individual study results and the narrative synthesis.

Data Synthesis

Because of substantial clinical and methodological heterogeneity between the included studies, a quantitative meta-analysis was not performed. Studies were synthesised narratively according to the principal clinical questions, including digital arterial revascularisation, primary arterial repair versus interposition vein grafting, consequences of unrepaired digital arterial injury, arterial margin assessment, single versus dual arterial reconstruction, and artery-only digital replantation. Differences in patient populations, injury mechanisms, anatomical levels, reconstructive techniques and outcome definitions were considered when interpreting findings. Effect estimates and statistical significance reported by the original studies were presented where available. Risk of bias assessments were incorporated into the interpretation of individual study findings. No pooled effect estimates, formal heterogeneity statistics, subgroup meta-analyses or publication bias analyses were performed because the number and heterogeneity of studies were insufficient for meaningful quantitative synthesis.

Registration 

No formal review protocol was registered for this systematic review, and no PROSPERO registration was obtained. The systematic review followed PRISMA 2020 guidelines. 

Results

Study Selection Process

The study selection process was undertaken in accordance with the PRISMA 2020 guidelines. The electronic database search identified 98 records, including 25 from PubMed or MEDLINE, 24 from Embase, 35 from Scopus, and 14 from the Cochrane Library. After removing 20 duplicate records, 78 unique records remained for title and abstract screening. Of these, 60 were excluded as they did not satisfy the predefined eligibility criteria. Consequently, 18 reports were identified for retrieval. However, one could not be obtained, resulting in 17 full-text reports being assessed for eligibility. Following full-text assessment, 11 reports were excluded: four case reports, one animal study, one editorial, two conference abstracts, and three reports published in languages other than English. Ultimately, six studies met all inclusion criteria and were included in the final systematic review and qualitative synthesis. The full process of study identification, screening, eligibility assessment, and inclusion is illustrated in the PRISMA 2020 flow diagram (Figure 1).

**Figure 1:**
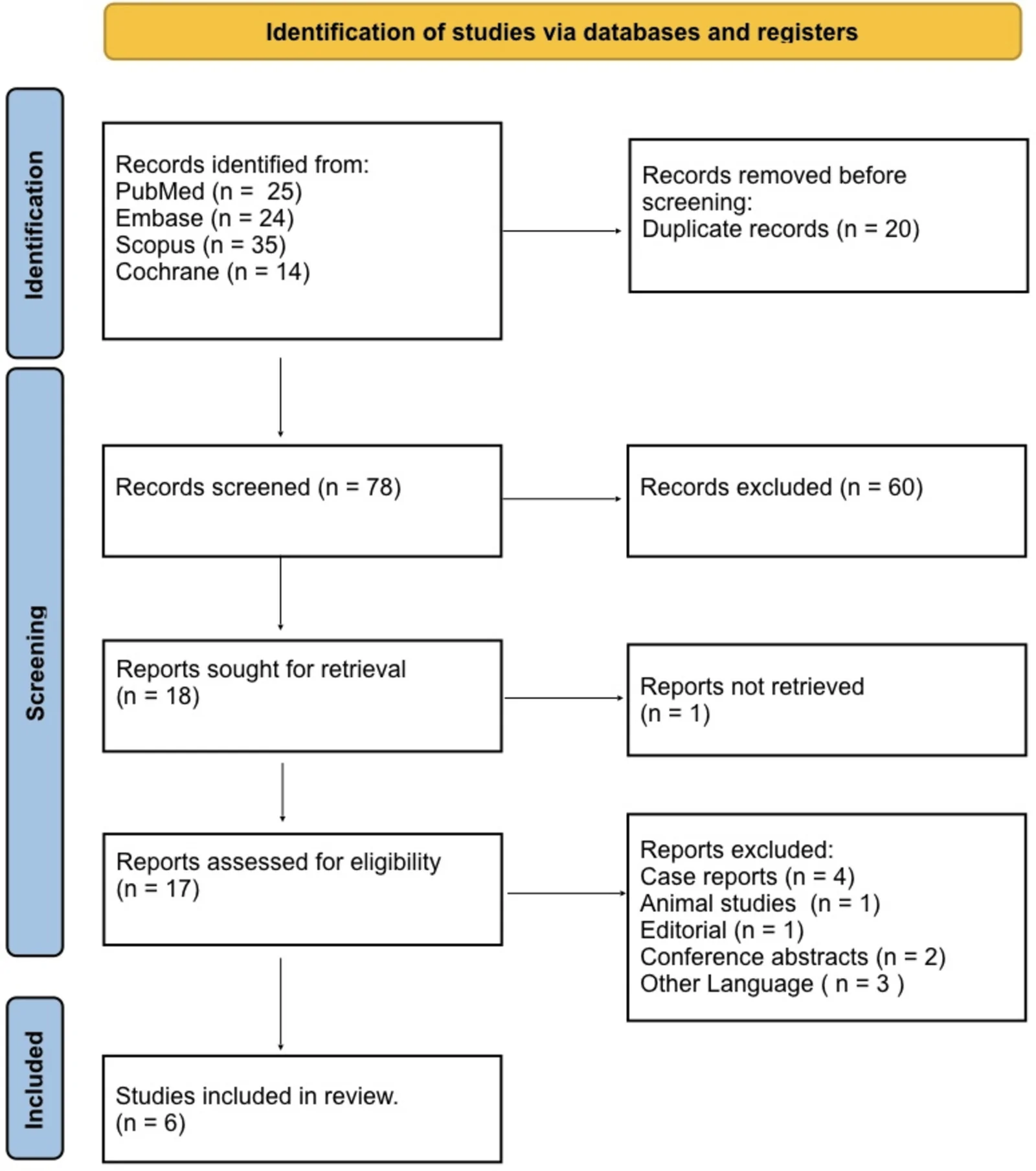
PRISMA 2020 Flow Diagram PRISMA: Preferred Reporting Items for Systematic Reviews and Meta-Analyses

*Characteristics of the Selected Studies* Table 2 shows six included studies comprising predominantly retrospective observational cohorts, with one prospective single-centre study. The studies addressed different but clinically complementary aspects of traumatic arterial injury of the hand, including paediatric digital arterial revascularisation, arterial reconstruction using interposition grafts, the clinical consequences of unrepaired digital artery injury, management of injured arterial ends, single versus dual arterial reconstruction, and artery-only digital replantation. Consequently, substantial clinical heterogeneity existed with respect to age, mechanism of injury, anatomical level, injury severity, reconstructive technique and outcome definitions. This heterogeneity precluded meaningful pooling of all studies into a single quantitative meta-analysis.

**Table 2:** Characteristics of the Included Selected Studies 2PD: Two-Point Discrimination; BMRC: British Medical Research Council; FLIR: Forward-Looking Infrared; QoL: Quality of Life; ED: Emergency Department; mm: Millimetres; p: p-value/probability value; %: Percentage; n: Number of participants or digits; vs: Versus; IQR: Interquartile Range; SD: Standard Deviation; CI: Confidence Interval; OR: Odds Ratio; RR: Relative Risk; Tamai I-III: Tamai classification zones I-III for levels of digital amputation; QoL: Quality of Life; US: Ultrasonography

Author & year	Population (P)	Study design/evidence level	Mechanism/arterial injury	Assessment	Intervention (I)	Comparator (C)	Primary outcome	Secondary outcomes	Statistical analysis/effect estimates	Follow-up	Clinical implication
Linnaus et al., 2016 [8]	25 children with traumatic digital arterial injury; injuries proximal to digits excluded	Retrospective cohort; descriptive study	Blunt 12/25 (48%) and penetrating 13/25 (52%) digital trauma	Clinical assessment of digital perfusion	Primary arterial repair, vein graft, thrombectomy; amputation in 1	Different operative approaches	Successful digital revascularisation/long-term sequelae	Complications, amputation, functional outcome	Descriptive statistics. Primary repair 15/25 (60%); vein graft 7/25 (28%); thrombectomy 1/25 (4%); amputation 1/25 (4%). Complications 12/25 (48%); long-term sequelae 2/25 (8%)	>24 weeks for long-term outcome	Attempt revascularisation when physical examination demonstrates inadequate perfusion; vein graft is an acceptable alternative when primary repair is not feasible.
Yildiran et al., 2019 [9]	48 patients with unilateral zone-II digital nerve injury; 24 with unrepairable digital artery injury and 24 with intact artery	Retrospective comparative cohort	Digital artery injury associated with finger laceration/zone-II nerve injury	Clinical vascular and sensory assessment	Digital nerve repair with artery left unrepaired in the arterial-injury group	Intact digital artery group	Sensory recovery	2-point discrimination, neuroma, cold intolerance, BMRC sensory grade	Mean 2PD 5.37 mm vs 5.29 mm; 2 neuromas vs 1; no statistically significant difference in 2PD, neuroma or cold intolerance	Mean 17.7 months	In a well-perfused finger with alternative arterial supply, failure to repair one digital artery did not worsen measured long-term sensory outcomes. Authors nevertheless recommend anatomical arterial repair when feasible.
Lee et al., 2019 [10]	127 patients undergoing digital revascularisation or replantation	Retrospective comparative cohort; Therapeutic III	Sharp, crush and avulsion digital trauma	Clinical + operative microsurgical assessment	Interpositional arterial vein graft	Primary arterial repair	Digit survival	Injury mechanism, graft requirement	Revascularisation survival: 91.5% vs 91.5% for graft vs primary repair. Replantation survival 48.1% vs 46.2%, p=0.88. Grafting more frequent in crush 62.5% and avulsion 72.2% than sharp injury 11.1%; RR 5.6, p=0.01 and RR 6.5, p=0.006, respectively. Regression found no association between graft use and digit survival	Study period 2007–2016	A required interposition graft should not be considered a contraindication to digital revascularisation/replantation. Particularly useful for crush/avulsion injuries.
Pichonnat et al., 2024 [11]	104 patients with acute traumatic arterial injury of the hand and/or wrist	Prospective single-centre multidisciplinary cohort; Level II	Laceration, crush, amputation, blast and power-tool injuries	Clinical assessment; microscopic histology of arterial margins; postoperative ultrasound	Trimming of proximal/distal arterial ends before anastomosis	Comparison according to mechanism and degree of arterial damage	Anastomotic/patency failure	Histological arterial damage; mechanism; clinical failure	42% arterial margins macroscopically damaged; 74% histologically damaged. Clinical failure 7.8%; patency failure 10.4%. Power-tool amputation associated with failure OR 8.19, p<0.01. Glass laceration associated with more normal histology OR 3.72, p<0.05. Shorter arterial resection associated with failure p<0.02	12 months	Do not assume the apparently healthy arterial end is normal. Trim damaged arterial segments before anastomosis; authors recommend >2 mm trimming generally and >4 mm for blast/amputation injuries, with vein grafting when necessary.
Tamulevicius et al., 2025 [12]	31 patients following complex traumatic hand injury in emergency department	Retrospective follow-up cohort	Complex finger injury requiring replantation	Clinical examination + FLIR thermal imaging + laser-speckle perfusion + sensory/functional testing	Reconstruction of one vs both proper digital arteries	Single vs dual arterial supply	Objective finger perfusion	Temperature, sensation, pain, grip strength, function/QoL	Comparative statistical analysis demonstrated better objective perfusion with dual arterial supply, while functional outcomes did not show a clear equivalent advantage	Long-term follow-up	Dual arterial reconstruction may improve perfusion, but one adequate digital artery can potentially provide satisfactory functional results in selected fingers.
Kobayashi et al., 2026 [13]	177 patients undergoing traumatic replantation; Tamai zones I–III; Emergency department presenting population	Retrospective cohort; Therapeutic Level III	Traumatic digital amputation	Clinical + microsurgical assessment; arterial characteristics; warm ischaemic time	Artery-only replantation	Arterial + venous replantation	Digit survival/replantation success	Injury level, arterial diameter, vein grafting, transfusion	Overall success 80.5% (165/205); zone I 84.4%, zone II 84.1%, zone III 67.4%. No statistically significant difference between artery-only and non-artery-only groups overall or by zone	2000–2024 cohort; follow-up reported in study	Venous anastomosis should not be pursued excessively, particularly in fingertip injuries; artery-only replantation can be successful in selected cases.

Risk of Bias Assessment

The six included studies demonstrated overall moderate to moderate-high risk of bias, primarily related to retrospective study designs, non-randomised treatment allocation, potential confounding, small sample sizes and heterogeneity in injury characteristics. The prospective study had a comparatively lower overall risk of bias but remained limited by its single-centre observational design and lack of a randomised comparator. Detailed domain-level assessments and the principal methodological limitations of each study are presented in Table 3. These limitations were considered when interpreting the comparative findings and the strength of the clinical implications.

**Table 3:** Bias Assessment of Included Studies through the JBI Scale JBI: Joanna Briggs Institute

Study	Selection/allocation bias	Confounding	Intervention classification	Outcome measurement	Missing data/follow-up	Statistical validity	Selective reporting	Overall risk of bias	Key methodological limitation
Linnaus et al., 2016 [8]	Moderate	Moderate-High	Low	Low	Moderate	Moderate	Low	Moderate	Retrospective design, small paediatric cohort, heterogeneous injury mechanisms and treatment strategies, and potential treatment-selection bias.
Yildiran et al., 2019 [9]	Moderate	High	Low	Low-Moderate	Moderate	Moderate	Low	Moderate-High	Non-randomised comparison between patients with unrepaired and intact digital arteries; associated digital nerve injury and other clinical factors may have introduced residual confounding.
Lee et al., 2019 [10]	Moderate	High	Low	Low	Moderate	Low-Moderate	Low	Moderate	Treatment allocation was not randomised and was influenced by the injury mechanism and arterial defect characteristics; retrospective data collection may have resulted in incomplete adjustment for confounders.
Pichonnat et al., 2024 [11]	Low–Moderate	Moderate	Low	Low	Low-Moderate	Low-Moderate	Low	Low-Moderate	Single-centre observational design, heterogeneous traumatic injuries and mechanisms, and absence of a randomised comparator; treatment and arterial resection decisions may have been influenced by injury severity.
Tamulevicius et al., 2025 [12]	Moderate	High	Low	Low	Moderate	Moderate	Moderate	Moderate-High	Small cohort, retrospective design and potential selection bias; objective perfusion findings may not directly translate into equivalent patient-reported or functional outcomes.
Kobayashi et al., 2026 [13]	Moderate	High	Low-Moderate	Low	Moderate	Moderate	Moderate	Moderate-High	Treatment selection was non-randomised and influenced by injury characteristics; substantial heterogeneity in injury level and mechanism may have introduced confounding when comparing artery only with arterial and venous replantation.

Digital Arterial Revascularisation

Linnaus et al. evaluated 25 paediatric patients with traumatic digital arterial injury in a retrospective trauma registry study [8]. The median age was 6.8 years, and both blunt and penetrating mechanisms were represented. All patients underwent operative management. Primary arterial repair and vein grafting were the principal reconstructive strategies, while thrombectomy and amputation were used in individual cases. Twelve patients (48%) experienced complications during initial follow-up; however, only two patients (8%) had long-term sequelae beyond 24 weeks. The authors concluded that digital arterial revascularisation in children can produce favourable functional outcomes and that primary repair is effective when technically feasible, with vein grafting providing an alternative when direct repair cannot be achieved. These findings support the principle that perfusion status should determine the urgency of intervention, particularly in an ischaemic digit. However, the evidence is limited by the small sample size, retrospective design and paediatric population, restricting direct extrapolation to adults.

Primary Arterial Repair Versus Interposition Vein Grafting

Lee et al. provided the largest comparative cohort among the included studies, comprising 127 patients with 171 affected digits, including 118 digital revascularisations and 53 digital replants [10]. Interposition grafts were used in approximately half of the revascularisations and replantations. In digital revascularisation, digit survival was 91.5% following both primary repair and graft repair, with no significant difference between the techniques. In replantation, survival was 48.1% with graft repair compared with 46.2% with primary repair, with no statistically significant difference (p = 0.88). Regression analysis demonstrated no association between interpositional graft use and digit survival. Importantly, graft use was strongly associated with the mechanism of injury. Grafting was used in 62.5% of crush injuries and 72.2% of avulsion injuries, compared with only 11.1% of sharp lacerations. The relative risk of requiring a graft was 5.6 for crush injury (p = 0.01) and 6.5 for avulsion injury (p = 0.006) compared with sharp laceration. The findings indicate that interposition vein grafting should not be interpreted as an inferior reconstructive strategy or as a contraindication to revascularisation. Rather, graft use appears to reflect the severity and extent of the zone of arterial injury.

Effect of Digital Arterial Injury on Sensory Outcome

Yildiran et al. reported mean two-point discrimination of 5.37 mm in patients with unrepairable digital artery injury compared with 5.29 mm in patients with an intact digital artery, with no statistically significant difference reported [9]. Sensory recovery according to the Medical Research Council classification was also comparable. These findings suggest that, in a selected well-perfused digit, an isolated unrepaired digital artery may not independently determine long-term sensory recovery. However, the study evaluated digital nerve outcomes rather than digit viability and therefore should not be interpreted as evidence against arterial reconstruction in an ischaemic digit.

Arterial Margin Assessment and Mechanism of Injury

Pichonnat et al. conducted a prospective single centre study of 104 patients with acute traumatic arterial injury of the hand and/or wrist, with 12-month follow-up [11]. Histological examination demonstrated arterial injury in 74% of specimens, despite the macroscopic appearance of the arterial margins. The frequency of histological damage varied substantially according to mechanism: 100% in blast injuries, 92% in mechanical lacerations, 82% in power tool lacerations, 80% in mechanical amputations, 67% in power tool amputations, 50% in glass lacerations and 33% in crush injuries. The overall clinical failure rate was 7.8%, while the arterial patency failure rate was 10.4%. Power tool amputation was significantly associated with anastomotic failure (OR 8.19; p < 0.01), while shorter arterial resection was also associated with failure (p < 0.02). Histologically normal arterial margins were more likely following glass laceration (OR 3.72; p < 0.05). These findings demonstrate that the mechanism of injury is an important determinant of arterial wall damage and may influence the length of arterial resection required before reconstruction.

Single Versus Dual Digital Arterial Reconstruction

Tamulevicius et al. retrospectively evaluated 31 patients following finger amputation associated with complex hand injury [12]. Patients were assessed following reconstruction of either one or both proper palmar digital arteries. Objective perfusion was evaluated using thermal imaging and laser speckle contrast analysis, while functional, sensory, pain, grip-strength and quality of life outcomes were also assessed. Patients undergoing dual artery reconstruction demonstrated significantly higher finger surface temperature, indicating improved peripheral perfusion. Laser speckle analysis also favoured dual reconstruction, although the difference did not reach statistical significance. Importantly, overall functional and quality of life outcomes were similar between groups, with the principal functional difference being improved temperature sensation in the dual artery group. Thus, the available evidence suggests that reconstruction of both digital arteries may provide better objective perfusion, but current evidence does not establish that dual reconstruction is necessary to achieve satisfactory overall functional outcomes when one reconstructed artery provides adequate perfusion.

Artery-Only Digital Replantation

The most recent included study, by Kobayashi et al., evaluated 177 patients involving 207 digits undergoing replantation of Tamai zones I-III between 2000 and 2024 [13]. Patients undergoing artery-only replantation were compared with those undergoing both arterial and venous reconstruction. Overall, 205 digits were available for analysis, with a survival rate of 80.5% (165/205). Survival was 84.4% in Tamai zone I, 84.1% in zone II and 67.4% in zone III. No statistically significant difference in survival was identified between artery-only and non-artery-only replantation groups, either overall or within individual Tamai zones. These findings provide evidence that artery-only replantation may be a viable option in selected distal digital amputations, particularly where reliable venous anastomosis is technically difficult. Nevertheless, the retrospective design and treatment-selection differences between groups limit the strength of this conclusion.

Discussion 

The available evidence supports a perfusion-driven approach to traumatic arterial injuries of the hand and digits, in which the need for reconstruction is determined primarily by distal tissue viability, the extent of arterial disruption, and the presence of reliable collateral circulation. Across the included studies, revascularisation was associated with satisfactory digit survival when perfusion was threatened, whereas an isolated digital arterial injury did not consistently result in adverse outcomes when adequate alternative arterial supply was maintained. This distinction is clinically important because the presence of an arterial injury alone does not establish critical ischaemia. Conversely, disruption of multiple arterial channels, extensive tissue injury, crush or avulsion mechanisms, and traumatic amputation may compromise perfusion and increase the need for urgent reconstruction. The evidence therefore supports prioritising restoration of perfusion in threatened or ischaemic digits while avoiding the assumption that every isolated digital arterial injury requires repair [8,10]. When arterial reconstruction is indicated, the choice of technique should be guided by the extent and nature of the arterial defect. Primary repair remains appropriate when the injured arterial ends can be approximated without tension, whereas interposition vein grafting provides an effective alternative when segmental arterial loss or tissue damage prevents direct anastomosis. Comparative evidence did not demonstrate inferior digit survival with vein grafting compared with primary repair, despite grafts being used more frequently in crush and avulsion injuries. This suggests that the need for a graft is more likely to reflect the severity and extent of the arterial injury than an intrinsically poorer reconstructive outcome. The mechanism of injury should therefore influence intraoperative assessment, particularly in crush, blast, power tool and amputation injuries, where arterial damage may extend beyond the visibly injured segment. The finding that apparently normal arterial margins may demonstrate microscopic structural damage further supports excision of compromised vessel until satisfactory arterial ends are obtained before reconstruction. Excessive resection should nevertheless be avoided because loss of arterial length may increase the need for grafting and may itself adversely affect reconstruction [10,11].

The evidence also indicates that restoration of arterial continuity should be balanced against the clinical consequences of additional reconstruction. In a well-perfused digit with an alternative arterial supply, isolated digital artery injury did not produce a demonstrable adverse effect on long-term sensory recovery in the available comparative evidence. This finding should not be interpreted as evidence that arterial repair is unnecessary in an ischaemic digit, but rather that reconstruction may be selectively individualised when collateral perfusion is demonstrably adequate. Similarly, reconstruction of both proper digital arteries may improve objective measures of peripheral perfusion compared with restoration of a single arterial supply, although this physiological benefit has not translated consistently into superior functional, sensory or quality of life outcomes. Thus, where one reconstructed artery provides reliable perfusion, dual arterial reconstruction should not automatically be considered essential in every case. The decision should instead incorporate the level and severity of injury, tissue viability, technical feasibility and associated tendon, nerve and skeletal damage [9,12]. Replantation represents a further situation in which the balance between anatomical reconstruction and clinical outcome is particularly important. The available evidence suggests that artery-only replantation can achieve acceptable survival in selected distal digital amputations when venous anastomosis is technically difficult or impractical. However, these findings should not be interpreted as establishing artery-only replantation as equivalent to conventional arterial and venous reconstruction in all circumstances. Injury level, mechanism, tissue damage, arterial calibre, ischaemia time and the technical feasibility of venous repair remain important determinants of outcome. Overall, the evidence supports a pragmatic approach in which venous reconstruction is pursued when feasible, while artery-only replantation may be considered selectively when the anticipated benefit of prolonged or technically difficult venous reconstruction is limited [13].

From a clinical management perspective, these findings support early assessment of digital perfusion using clinical examination, including colour, temperature, capillary refill and Doppler signals where appropriate, followed by urgent specialist assessment when perfusion is threatened. Revascularisation should be prioritised for an ischaemic or threatened digit when technically feasible. For an arterial injury requiring reconstruction, primary repair is preferred when a tension-free anastomosis can be achieved, while an interposition vein graft is appropriate for a segmental defect that prevents direct approximation. In adequately perfused digits with dependable collateral circulation, isolated digital arterial injury may be managed without reconstruction in selected circumstances, although the decision should consider the overall injury pattern rather than the artery in isolation. Distal amputations requiring replantation may be considered for artery-only reconstruction when venous anastomosis is technically unsuitable. These recommendations should be regarded as evidence-informed principles rather than a validated emergency department algorithm because none of the included studies directly evaluated a standardized emergency assessment, imaging or referral pathway. The evidence base has several important limitations. Only six studies met the inclusion criteria, and most were retrospective observational cohorts with relatively small or heterogeneous populations. Treatment allocation was non-randomised and was frequently influenced by injury mechanism, tissue damage, anatomical level and intraoperative findings, creating substantial potential for selection bias and residual confounding. Outcome definitions also varied considerably, ranging from digit survival and arterial patency to sensory recovery, objective perfusion and patient-reported function, limiting direct comparison between studies. The paediatric population in one study further restricts generalisation to adults, while the inclusion of replantation cohorts introduces clinical circumstances that differ from isolated arterial laceration. The overall certainty of the available evidence is therefore limited, and the reported associations should not be interpreted as definitive comparative treatment effects.

Future research should prioritise prospective multicentre studies using standardised definitions of arterial injury, perfusion status, reconstructive technique and outcome. Studies should directly compare primary repair with interposition grafting where clinically appropriate and should account for injury mechanism and severity when evaluating reconstructive outcomes. Comparative studies of single versus dual digital arterial reconstruction should incorporate both objective perfusion measures and clinically meaningful outcomes such as digit survival, sensory recovery, cold intolerance, range of motion, grip strength and patient-reported function. Further prospective evaluation of artery-only versus conventional arterial and venous replantation is also required, particularly across different levels of digital amputation. Finally, development and validation of a hand-specific emergency department assessment and referral pathway would help determine how clinical examination, Doppler assessment and vascular imaging should be integrated into the early management of these injuries.

## Conclusions

Traumatic arterial injuries of the hand and digits require assessment of digital perfusion and the extent of vascular injury. The available evidence suggests that an isolated digital arterial injury may not invariably require reconstruction when adequate collateral perfusion is maintained. Primary repair may be used when a tension-free anastomosis is achievable, while interposition vein grafting provides an option for segmental arterial defects. Dual arterial reconstruction may improve objective perfusion, although a consistent functional advantage has not been demonstrated. Artery-only replantation has also demonstrated acceptable survival in selected distal digital amputations. These findings are derived from six heterogeneous studies, predominantly retrospective observational cohorts, and should therefore be interpreted cautiously because of potential confounding, selection bias and limited comparative evidence.
